# Narrative Expertise in Oncology: An Integrated Training Model to Advance the Field

**DOI:** 10.2196/78010

**Published:** 2025-10-03

**Authors:** Trisha K Paul, Erica C Kaye

**Affiliations:** 1 Department of Pediatrics, Division of Hematology/Oncology, University of Rochester Medical Center, 601 Elmwood Ave, Box 777, Rochester, NY, 14642, United States, 1 585-275-2981; 2 Department of Health Humanities and Bioethics, University of Rochester School of Medicine and Dentistry, Rochester, NY, United States; 3 Department of Oncology, St. Jude Children's Research Hospital, Memphis, TN, United States

**Keywords:** narrative oncology, cancer care, pediatric oncology, physician-writer, creative writing, narrative medicine, writing, training, education

## Abstract

Despite growing evidence that narrative expertise may benefit cancer care professionals and the field, few hematology-oncology trainees pursue graduate degrees in the humanities. For those trainees with a particular interest in humanism in medicine, we advocate for integration of a Master of Fine Arts (MFA) degree concurrent with fellowship training. This pathway enables trainees to gain advanced skills in narrative competence, informing research and scholarly activities during fellowship and building a foundation for future careers that promote humanism in the field of hematology-oncology across clinical practice, education, research, and advocacy. Narrative competence describes the ability to create space for and elevate the voices of patients, families, and clinicians, which includes active listening, reflecting, sharing, and being moved by stories. In this paper, we review evidence suggesting that frequent exposure to suffering can threaten career longevity for cancer care clinicians, and we highlight narrative competence as an approach to mitigate moral distress, improve well-being, and bolster resilience for our workforce. The influence of narrative competence extends beyond patient care, with meaningful ramifications for advancing research, education, and advocacy efforts across the field. We encourage institutions with hematology-oncology fellowship programs that have capacity to support graduate studies to include the MFA as an option for trainees who aim to become thought leaders and experts in narrative competence. The MFA serves as a strategic mechanism to invest in growing the next generation of hematologist-oncologists with expertise in narrative competence to advance the field.

## Background

Hematology-oncology fellowship aims to prepare the next generation of physicians committed to excellence in clinical practice, research, education, advocacy, and policy to advance cancer care. Medical and pediatric oncology training traditionally comprises one clinically-focused year followed by two years of dedicated research. Often, trainees pursue graduate degrees concurrent with their research years [1], such as a Doctor of Philosophy (PhD) for basic scientists or a Master of Public Health for clinical researchers. Increasingly, nontraditional degrees in business, health care administration, health policy, ethics, and medical education are integrated within fellowship [1]. Graduate degrees in the humanities, however, remain uncommon, despite growing evidence that expertise in narrative competence may benefit oncology professionals and the field [2,3].

Narrative competence refers to the ability to create space for and elevate the voices of patients, families, and clinicians through active listening, reflecting, sharing, finding meaning in, and being moved by stories [2,4-6]. Through narrative competence, clinicians can deepen compassion and bolster therapeutic relationships in the context of stressful encounters [7]. Frequent exposure to suffering increases burnout and threatens career longevity for oncology clinicians [5], and narrative competence offers a humanistic approach to mitigate moral distress, improve wellbeing, and bolster resilience for our workforce [8-11].

## Rationale for Supporting Narrative Competence in Oncology Training

The influence of narrative competence extends beyond patient care, with meaningful ramifications for advancing research, education, and advocacy [12,13]. For example, narrative elicitation in research can elevate voices from patients and families, and effective storytelling can strengthen grant applications and streamline dissemination of findings to promote scientific discovery, collaboration, and activism [14]. Clinician narratives about cancer can educate the public about cutting-edge preventative, therapeutic, and supportive care strategies [12,14]. Storytelling exercises in educational curricula fostered solidarity among trainees and increased reported preparedness to navigate work-life balance [9]. Strategic marketing through storytelling propels advocacy to mitigate health disparities, as seen in communications by the Global Initiative for Childhood Cancer [13]. Narratives can function as a lobbying tool for policy design and implementation and for gaining public support to improve cancer care [15].

The importance of how we tell stories about cancer and how these stories can catalyze change begs the question: how can we promote expertise in narrative-based practice for oncology professionals? Currently, few fellowships offer formal instruction in writing or other forms of narrative-based practice, limiting skill development during training. Growing the next generation of oncology clinicians with narrative expertise could have far-reaching implications for advancing the field. Here, we describe a novel track for graduate-level education in creative writing concurrent with medical subspecialty training to promote narrative competence among physicians and advance the field of narrative in oncology.

## Creative Writing as a Flexible Graduate Degree Option for Oncologists

For hematology-oncology trainees with a particular interest in humanism in medicine, we advocate for integration of a Master of Fine Arts (MFA) degree concurrent with fellowship training. The MFA in Creative Writing is a graduate degree that offers students the opportunity to hone their writing craft and lifelong careers as writers with guidance and personalized feedback from expert writing instructors and published authors as faculty. This pathway can equip trainees with advanced skills in narrative competence, informing research and other scholarly activities during fellowship and building a foundation for a future career that promotes humanism in clinical care, education, research, and advocacy.

While traditional MFAs comprise two to three years of in-person education, ‘low-residency’ MFA programs offer two-year part-time graduate degrees that can be completed alongside full-time training. Presently, 52 low-residency MFA programs exist in the United States, offering a hybrid model of virtual asynchronous learning combined with in-person didactics [16]. Most MFA programs include tracks in nonfiction that can be tailored to the academic goals of oncology trainees and professionals. Formal training through an MFA provides infrastructure to develop advanced skills in narrative competence and writing through experiential learning, practical skill development, and personalized one-on-one feedback from renowned writers and scholars. While opportunities to enhance narrative skills exist outside of the MFA through nonaccredited classes and online writing communities, graduate-level training provides a comprehensive foundation in narrative techniques with longitudinal mentorship tailored to the student’s content area of interest. Additionally, each degree culminates in a full-length manuscript through a master’s thesis, readily translatable into scholarly writing for scientific or lay communities to advance the field.

## Pursuit of an MFA Degree During Oncology Training

**Figure 1.**
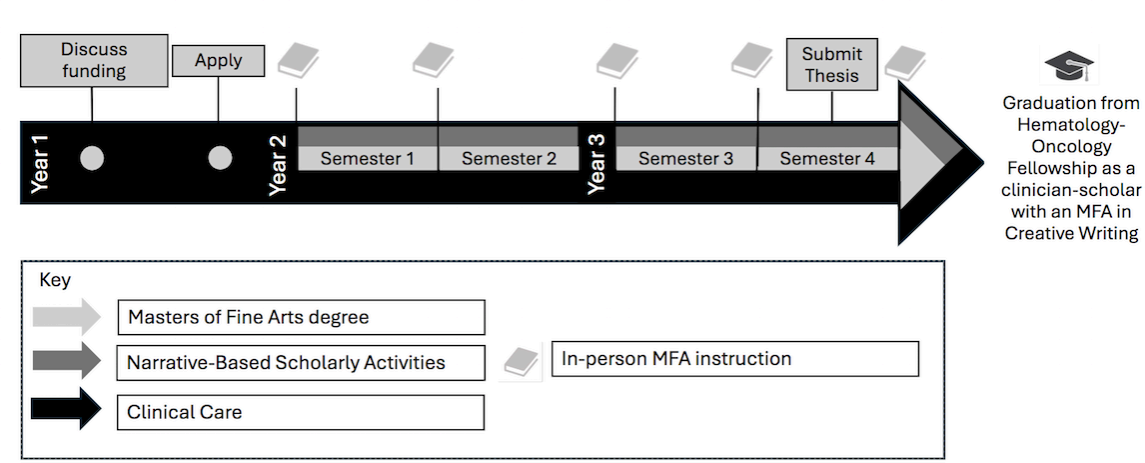
An embedded training model for pursuit of a Master in Fine Arts (MFA) degree in creative writing during Hematology/Oncology Fellowship. During the first year of hematology-oncology fellowship, trainees should engage in conversations about graduate-level funding with their institution. Each semester of a low-residency MFA begins with a full-time residency, comprising 7‐10 days of in-person instruction. Students participate in a total of 5 residencies over the 4 semesters, with the fifth and final residency occurring after submission of a thesis. Joint pursuit of the MFA during hematology-oncology fellowship can promote narrative skills that can inform a trainee’s scholarly activities in research, education, and advocacy. MFA: Master of Fine Arts.

To our knowledge, St. Jude Children’s Research Hospital is the first institution in the United States to offer a formal track for fellows to integrate an MFA into their training, combining protected research time with pursuit of this degree (Figure 1). Recognizing that common barriers to pursuit of an MFA include time and cost, our institution subsidized the MFA similar to other graduate degrees. Our first trainee to complete an MFA through the integrated narrative oncology track demonstrated unique achievements in scholarly productivity across research, clinical care, education, and advocacy domains, supporting the feasibility of completing an MFA within the fellowship period. The trainee received targeted education and feedback to enhance her narrative competence and writing skills, resulting in publication of numerous essays in impactful journals including the Journal of the American Medical Association [17]. With research mentorship through fellowship and writing techniques learned through the MFA, the trainee also designed, implemented, and rigorously studied a narrative writing intervention for adolescents and young adults with cancer that was found to be feasible, acceptable, and impactful. The trainee further conducted a comprehensive systematic review of the literature, synthesizing the benefits of narrative-based interventions for cancer care professionals. During her MFA, the trainee also developed and implemented narrative-based educational programming for interdisciplinary fellows across multiple hospitals, including integration of narrative approaches into longitudinal curricula, workshops, and bedside rounding. Additionally, the trainee taught narrative competence to undergraduate, medical, and healthcare students, as well as connected with multidisciplinary cancer care professionals nationally and internationally to foster community and collaboration related to narrative competence across the field. Currently, the trainee is orchestrating efforts to create a national consortium to leverage narrative hematology-oncology expertise and consolidate resources in a shared central location to advance research in narrative-based practice. From a scholarship perspective, the MFA promoted prolific scientific and narrative writing within the 24 month period of the MFA, including four original research manuscripts [4,18,19,20], two narrative essays in peer-reviewed medical journals [17, 21], an essay in a book [22], and three lay publications raising awareness about narrative writing in cancer care [23-25].

Figure 1 describes a novel training pathway for hematology-oncology fellows with a targeted interest in developing expertise in narrative competence. Hematology-oncology fellowship traditionally includes one year of clinical medicine followed by two years of protected scholarship time. MFA applications typically are due in the spring and consist of a personal statement, letters of recommendation, and a 20-page writing sample. The embedded training model described in Figure 1 enables pursuit of graduate-level training to hone expertise in narrative competence with concurrent impact in clinical practice and scholarly activities during training and beyond.

Positioning trainees to be thought leaders in narrative oncology as faculty members can advance narrative-based clinical care, education, research, and advocacy in cancer care in alignment with individual trainee interests. Further quantitative and qualitative data are needed to evaluate how participation in an MFA in creative writing during fellowship may impact metrics like trainee wellness, career development, and patient-centered outcomes. Measurable deliverables might include frequency and extent of didactics and experiential learning opportunities related to narrative oncology, volume of students with exposure to the field and access to mentorship in this area, and growth of narrative-based research studies conducted by graduates of the narrative oncology track.

We believe that institutions with fellowship programs that have capacity to support graduate studies should include the MFA as an option for hematology-oncology fellows who aim to be thought leaders in narrative medicine. We also encourage institutions to anticipate logistical aspects to streamline this process. As with other graduate programs in the United States, incoming trainees should apply during the spring of their first year to align coursework with protected research time. Trainees accepted into an MFA program may need to proactively arrange their call schedules to accommodate 1‐2 weeks spent at in-person residencies each semester. Barriers for trainees to receive support for the MFA across diverse fellowship settings may include limitations in clinical scheduling flexibility, lack of institutional precedent, lack of institutional awareness about the value of narrative expertise in oncology, or cost. For programs that subsidize the cost of graduate training for fellows, overall costs for an MFA are typically less than those for other master programs. Including costs for transportation and accommodations during in-person residencies, the cost of a low residency MFA generally ranges from US $30,000 to US $80,000, compared to traditional costs for other graduate degrees that often fall in the US $60,000‐ US $100,000 range. Potential return on investment from the MFA in creative writing may include advancement of the nascent field of narrative oncology at a national level; increased opportunities for networking, mentorship, career development, collaboration, and community-building in narrative oncology; and improvements in job satisfaction and trainee or faculty wellbeing. Growing awareness of narrative oncology and mentorship across institutions may help facilitate increased implementation of this training pathway across diverse fellowship settings.

In summary, the MFA serves as a strategic mechanism to invest in growing the next generation of oncologists with expertise in narrative competence. Storytelling is integral to advancing the field of oncology [2], and we encourage institutions to explore approaches to implementing this innovative track for trainees and faculty to gain the requisite skills to harness narrative-based practice as a powerful tool to advance the field through clinical care, research, education, and advocacy.

## Abbreviations:

MFA: Master of Fine Arts
