# Correction: Narrative Expertise in Oncology: An Integrated Training Model to Advance the Field

**DOI:** 10.2196/86071

**Published:** 2025-10-28

**Authors:** Trisha K Paul, Erica C Kaye

**Affiliations:** 1 Department of Pediatrics, Division of Hematology/Oncology, University of Rochester Medical Center, 601 Elmwood Ave, Box 777, Rochester, NY, 14642, United States, 1 585-275-2981; 2 Department of Health Humanities and Bioethics, University of Rochester School of Medicine and Dentistry, Rochester, NY, United States; 3 Department of Oncology, St. Jude Children's Research Hospital, Memphis, TN, United States

In “Narrative Expertise in Oncology: An Integrated Training Model to Advance the Field” [1], the authors noted three errors.

The corresponding author’s email address has been changed to the following:

trisha_k_paul@urmc.rochester.edu

In addition, reference 23 has been changed from:

23. Paul TK. Why share your childhood cancer story. Together Blog: Jude Children’s Research Hospital. Mar 19, 2024.

The reference now appears as:

23. Paul TK. Why share your childhood cancer story. Together Blog: St. Jude Children’s Research Hospital. Mar 19, 2024.

Finally, reference 22 has been changed from:

22. Paul TK. Goldstein MA, Tran KM, editors. Work on Professionalism” Becoming a Better Physician: Insightful and Inspirational Stories from Attending Physicians, Residents, and Medical Students. Springer; 2024:8-11. ISBN: 3031694120

The reference now appears as:

22. Paul TK. Goldstein MA, Tran KM, editors. “Work on Professionalism” Becoming a Better Physician: Insightful and Inspirational Stories from Attending Physicians, Residents, and Medical Students. Springer; 2024:8-11. ISBN: 3031694120

The correction will appear in the online version of the paper on the JMIR Publications website, together with the publication of this correction notice. Because this was made after submission to PubMed, PubMed Central, and other full-text repositories, the corrected article has also been resubmitted to those repositories.
